# Management of Recurrent Depressive Disorder, Anxiety, and Insomnia in a Patient With Philadelphia Chromosome-Positive Acute Lymphoblastic Leukaemia Receiving Dasatinib Therapy: A Case Report

**DOI:** 10.7759/cureus.117996

**Published:** 2026-10-15

**Authors:** Zanda Laura Zariņa, Ieva Everte

**Affiliations:** 1 Department of Psychiatry, Strenci Psychoneurological Hospital, Strenči, LVA; 2 Department of Residency, Riga Stradiņš University, Rīga, LVA

**Keywords:** acute lymphoblastic leukaemia, anxiety, dasatinib, depression, insomnia

## Abstract

Psychiatric symptoms, including depression, anxiety, and insomnia, are common among patients with cancer and may complicate the management of oncological disease. This report presents a case of a patient with Philadelphia chromosome-positive acute lymphoblastic leukaemia (ALL) receiving dasatinib treatment who experienced a recurrence of depressive symptoms accompanied by insomnia, severe anxiety, and fatigue. The case highlights the challenges of psychopharmacological management in the context of potential drug-drug interactions associated with concomitant oncological treatment.

A 55-year-old woman was admitted to a psychiatric inpatient unit because of worsening depressive symptoms, insomnia, severe anxiety, and fatigue. Pharmacological treatment was adjusted during hospitalisation, with treatment decisions influenced by potential pharmacokinetic interactions, QT interval prolongation risk, and the patient's concerns regarding medication changes. In addition to pharmacological treatment, the patient participated in a multidisciplinary psychiatric rehabilitation programme. During hospitalisation, her overall clinical condition gradually improved, with improvements in mood, anxiety, and sleep.

Individualised psychopharmacological treatment, careful consideration of potential drug-drug interactions, and collaboration between psychiatrists and oncologists are important in the management of psychiatric comorbidity in patients with ALL. Patient engagement in treatment decisions may further support a treatment approach tailored to individual needs.

## Introduction

Acute lymphoblastic leukaemia (ALL) is a haematological disease characterised by the clonal proliferation and accumulation of lymphoid progenitor cells in the bone marrow and extramedullary sites [1]. ALL is the most common cancer diagnosed in children, accounting for approximately 25% of cancer diagnoses among children younger than 15 years [2]. ALL exhibits a bimodal age distribution, with incidence peaks at approximately 5 and 50 years of age; around 11% of cases occur in adults aged 60 years or older [1]. ALL occurs slightly more frequently in males than in females, with age-adjusted incidence rates of 2.1 and 1.6 cases per 100,000, respectively [3]. Several factors have been associated with an increased risk of ALL, including previous chemotherapy or radiation therapy, exposure to high levels of ionising radiation, and certain genetic disorders [4].

Among adults with B-cell ALL, Philadelphia chromosome-positive (Ph+) disease represents the most common genetic subtype, with its incidence increasing with age [5]. Philadelphia chromosome-positive (Ph+) B-cell ALL has historically been considered a high-risk disease; however, the introduction of BCR:ABL1-targeted tyrosine kinase inhibitors (TKIs), including imatinib, dasatinib, and ponatinib, has substantially improved treatment outcomes and long-term survival in patients with Ph+ B-cell ALL [1,6].

Depressive disorders are highly prevalent worldwide and represent a substantial proportion of the global burden of mental health disorders, with higher rates observed among females [7]. Recurrent depressive disorder is characterised by repeated episodes of depression, with the severity of the current episode assessed based on the patient’s clinical presentation [8]. According to data from the Latvian Centre for Disease Prevention and Control, 13,794 adults aged 18 years and older were registered with mood (affective) disorders (International Classification of Diseases, Tenth Revision (ICD-10) codes F30-F39) in Latvia in 2024 [9]. According to a 2023 population-based study conducted in Latvia, the prevalence of clinically significant depressive symptoms among adults was 6.4%, with a higher prevalence among women than men (7.7% vs. 4.8%) [10].

Among adults with haematological malignancies, anxiety and depressive symptoms appear to be more prevalent than in the general population, particularly during active treatment, affecting 34% and 31.3% of patients, respectively, compared with 24.5% and 16.1% among those under watchful waiting [11]. Insomnia is a common sleep disorder among patients with cancer, occurring approximately three times more frequently than in the general population [12]. In addition to anxiety, depression, and sleep disturbances, patients with cancer may experience other symptoms, such as fatigue, appetite changes, and emotional distress. The management of psychiatric symptoms may be further complicated by potential drug-drug interactions between psychotropic and anticancer medications [13].

In this paper, we report the case of a 55-year-old woman with recurrent depressive disorder, anxiety, and severe insomnia in the context of Ph+ ALL receiving dasatinib therapy. This case highlights the challenges of individualised psychopharmacological management in the context of concomitant oncological treatment, particularly the need to consider potential drug-drug interactions, treatment tolerability, and the balance between clinical efficacy and safety.

## Case presentation

A 55-year-old woman was admitted to a psychiatric hospital following referral from her family doctor due to severe anxiety, insomnia, depressed mood, markedly reduced interest or pleasure in almost all activities, and fatigue.

History and presenting concerns

The patient's psychiatric and oncological history and treatment course are summarised in Table 1.

**Table 1:** Timeline of the patient's psychiatric and oncological history and treatment Years are presented relative to the patient's initial psychiatric presentation to preserve patient confidentiality. ALL: acute lymphoblastic leukaemia

Time	Psychiatric and oncological history and treatment
Year 1	First attended outpatient psychiatric care. Treatment for recurrent depressive disorder with fluvoxamine maleate 50 mg once daily and flupentixolum 1 mg twice daily was initiated and subsequently continued as long-term treatment.
Year 6	Diagnosed with ALL. Chemotherapy in combination with imatinib was initiated. The previously established psychiatric treatment was continued.
Year 8	Treated in the inpatient day department due to a recurrence of depressive symptoms. ALL was in remission, and she continued Imatinib as ongoing antineoplastic therapy.
Year 9	According to the patient's account, Philadelphia chromosome positivity was first identified during haematological follow-up, prompting further evaluation and subsequent adjustment of antineoplastic therapy. The patient's psychiatric condition gradually deteriorated, with persistent depressive and anxiety symptoms.
Year 10	Antineoplastic therapy was changed from imatinib to dasatinib 100 mg once daily.
Three weeks before psychiatric hospitalisation	The patient changed her outpatient psychiatrist. Pharmacological treatment was adjusted to escitalopram 10 mg in the morning, buspirone hydrochloride 5 mg twice daily, quetiapine 100 mg at night and bromazepam 1.5 mg for severe anxiety attacks. At that time, she continued the treatment prescribed by her oncologist: calcium 500 mg and vitamin D3 4200 IU in the morning, folic acid 2 mg in the morning, fish oil 1 capsule at noon, and dasatinib 100 mg in the morning.
Psychiatric hospitalisation	Admitted to the psychiatric inpatient unit due to worsening depressive and anxiety symptoms, insomnia, and fatigue. At admission, she was receiving dasatinib together with supportive treatment prescribed by her oncologist and was reported to be in ALL remission.

The patient was diagnosed with ALL at the age of 51. The patient reported no known significant exposure to ionising radiation and no history of previous chemotherapy or radiotherapy. There was no relevant family history of haematological or oncological malignancies. 

The subsequent clinical course was characterised by persistent depressive and anxiety symptoms, with worsening insomnia preceding psychiatric hospitalisation. At the time of admission, her main concerns were severe anxiety, insomnia, depressed mood, markedly reduced interest or pleasure in almost all activities, and fatigue. 

The oncological history was obtained primarily from the patient's account, as the original oncology and haematology records from the time of the initial ALL diagnosis were unavailable for review. Details of the initial disease characteristics and treatment course were, therefore, based on the patient's account and available subsequent medical documentation.

Mental state examination on admission

There were no signs of impaired or altered consciousness. The patient was alert and oriented. She was well-groomed and appropriately dressed. She was readily engaged in verbal contact and responded to questions appropriately and relevantly. She provided information, actively participated in the conversation, and asked the physician questions. At the time of admission, the patient reported sleep disturbances with frequent nocturnal awakenings and a total sleep duration of approximately two to three hours per night, low mood, anxiety, inner restlessness, difficulty concentrating, loss of interest and enjoyment in life, nervousness, inability to calm herself, and difficulty relaxing. During the interview, she appeared anxious and apprehensive. Her mood was depressed. Thought processes were coherent, sequential, and of normal rate, but were predominantly focused on her physical health and insomnia. She repeatedly expressed a strong desire to feel better as soon as possible. Attention was partially sustained. Cognitive and memory functions appeared grossly intact, although she subjectively reported difficulties with concentration. No psychotic symptoms were observed at the time of assessment. She denied suicidal ideation and plans to harm herself. Insight into her condition and critical judgment were preserved. 

Non-pharmacological and pharmacological treatment 

The pharmacological treatment adjustments made before and during hospitalisation are summarised in Table 2.

**Table 2:** Timeline of pharmacological treatment adjustments before and during hospitalization. The oncological treatment regimen was continued unchanged throughout hospitalization.

Time point	Changes in pharmacological treatment
Prior to hospitalisation	Escitalopram 10 mg in the morning; buspirone hydrochloride 5 mg twice daily; quetiapine 100 mg at night; bromazepam 1.5 mg as needed for anxiety
Ongoing oncological treatment*	Calcium 500 mg and vitamin D3 4200 IU in the morning, folic acid 2 mg in the morning, fish oil 1 capsule at noon, and dasatinib 100 mg in the morning
Day 1 (admission)	Escitalopram increased to 15 mg in the morning; buspirone hydrochloride increased to 10 mg three times daily; zopiclone 7.5 mg at night initiated; bromazepam 1.5 mg up to once daily as needed
Day 2	Quetiapine increased to 125 mg at night
Day 4	Zopiclone increased to 11.25 mg at night
Day 6	Escitalopram increased to 20 mg in the morning
Day 10	Quetiapine increased to 150 mg at night
Day 15	Patient requested a reduction in pharmacological treatment. Zopiclone decreased to 7.5 mg at night. Due to persistent anxiety, phenibut 250 mg twice daily initiated
Day 22	Phenibut decreased to 125 mg twice daily; quetiapine decreased to 125 mg at night
Day 26	Meldonium 500 mg once daily initiated

At admission, the treatment regimen was adjusted in response to persistent depressive, anxiety, and insomnia symptoms. Bromazepam was prescribed as needed for severe anxiety episodes, which were characterised by intense fear, inner restlessness, tremor, shortness of breath, and a pronounced sense of anxiety. The treatment regimen recommended by the oncologist was continued. Mirtazapine and trazodone were considered as potential treatment options but were not initiated. 

During the first days of hospitalisation, the patient continued to report sleep difficulties. On the second day, quetiapine was increased. The dose adjustment was considered acceptable given that the medication had been well tolerated up to that point. The patient was reluctant to switch to an alternative medication; therefore, continuation and dose adjustment of the existing treatment was preferred. 

On day four, persistent anxiety and sleep difficulties were characterised primarily by difficulty initiating sleep and repeated awakenings during the early part of the night. The patient also subjectively described inner restlessness, tension, and anxiety during the night, which further interfered with her ability to fall and remain asleep. Zopiclone was therefore increased.

By day six, the patient reported an improvement in sleep duration to approximately five to six hours per night; however, anxiety, depressed mood, and fatigue persisted. Escitalopram was, therefore, increased.

On day 10, sleep difficulties persisted, and quetiapine was further increased.

By day 15, the patient reported a slight improvement in her overall condition. However, she continued to experience anxiety symptoms and expressed concerns about becoming dependent on medication and requested a reduction in pharmacological treatment. Information regarding the potential risks of medication dependence and withdrawal was provided; nevertheless, she wished to reduce treatment. Consequently, zopiclone was decreased. Due to persistent anxiety symptoms, phenibut was subsequently initiated at a dose of 250 mg twice daily as a short-term, course-based treatment for up to four weeks. During treatment reduction with phenibut, the patient showed no signs of potential withdrawal symptoms.

On day 22, the patient reported further improvement and requested a reduction in phenibut and quetiapine. Both medications were subsequently reduced.

On day 26, meldonium was initiated to address the patient's persistent fatigue and reduced functional capacity, and overexertion. In this case, meldonium was used as symptomatic treatment.

Patient safety was monitored throughout hospitalisation. Routine laboratory tests were performed, including a complete blood count, liver and renal function tests, and urinalysis. ECG assessments performed before hospitalisation and prior to discharge showed no clinically significant abnormalities.

In addition to pharmacological treatment, throughout the hospitalisation, the patient participated in a multidisciplinary psychiatric rehabilitation programme involving physiotherapy, occupational therapy, music therapy, visual arts therapy, dance movement therapy, drama therapy, and psychological interventions. Psychological interventions were provided with a cognitive behavioural therapy (CBT) and supportive therapy orientation.

Clinical outcome and follow-up

During hospitalisation, the patient received pharmacological and non-pharmacological treatment. As no specific comprehensive guidelines are available for the pharmacological management of psychiatric symptoms in patients with concomitant cancer, treatment decisions were guided by the patient's clinical presentation, available evidence, and relevant national clinical guidance, including the Latvian Guidelines for the Diagnosis and Treatment of Depression [14]. Treatment decisions were individualised according to the patient's clinical presentation, concomitant oncological and psychotropic medications, potential drug-drug interactions, and the need for ongoing monitoring of treatment efficacy and safety. The patient demonstrated gradual clinical improvement, with a reduction in depressive and anxiety symptoms, improved mood, and improved sleep. Sleep duration increased from approximately two to three hours per night before admission to five to six hours per night during hospitalisation. On admission, the patient scored 18 points on the Patient Health Questionnaire-9 (PHQ-9) [15] and 11 points on the Generalized Anxiety Disorder-7 (GAD-7) [16], but at discharge, the scores had decreased to 9 on the PHQ-9 and 7 on the GAD-7, respectively. The patient subsequently requested discharge despite the treating physicians’ concerns and recommendation to continue inpatient treatment for an additional week. At discharge, the patient was in an improved clinical condition and was discharged with recommendations for continued outpatient psychiatric and haematological follow-up.

## Discussion

This case report describes a patient with recurrent depressive disorder and prominent anxiety symptoms who suffers from Philadelphia chromosome-positive acute lymphoblastic leukaemia receiving dasatinib therapy. An important limitation was the unavailability of the original oncology and haematology records from the time of the initial diagnosis, which prevented independent verification of the patient's initial disease characteristics and treatment history. This case also highlights a practical challenge encountered in clinical practice: obtaining a complete longitudinal medical history when historical records from different healthcare institutions are unavailable or cannot be readily accessed.

The patient required several psychopharmacological treatment adjustments during hospitalisation. Escitalopram is a selective serotonin reuptake inhibitor (SSRI) approved for the treatment of major depressive disorder and generalised anxiety disorder [17]. In this case, escitalopram was continued alongside dasatinib, which has the potential to prolong the QT interval; concomitant use may result in additive QT-prolonging effects, warranting caution and appropriate monitoring [18,19]. Baseline ECG showed sinus rhythm at 77 beats/min, with a PR interval of 144 ms, QRS duration of 101 ms, and QTc of 387 ms, without clinically significant abnormalities. Electrolyte abnormalities, particularly involving potassium, magnesium, and calcium, may further increase the risk of QT prolongation and cardiac arrhythmias. The Latvian clinical algorithm for the recognition, management, and treatment of depression in adults recommends considering electrolyte testing (sodium (Na), potassium (K), calcium (Ca), magnesium (Mg), and phosphate) in the presence of dehydration, excessive alcohol use, or endocrine disorders that may cause electrolyte disturbances [14]. None of these risk factors was documented in this patient, and electrolyte measurements were not performed during hospitalisation. This represents a limitation, as the contribution of electrolyte abnormalities to QT-prolongation risk could not be assessed. Escitalopram was selected as the main antidepressant and was gradually increased from 10 mg to 20 mg daily because of persistent depressive and anxiety symptoms. Although alternative antidepressant strategies were considered, the patient was reluctant to change escitalopram, which had only recently been initiated in the outpatient setting. The potential activating effects of escitalopram and its possible contribution to sleep disturbances were also considered when evaluating treatment options.

Mirtazapine and trazodone were considered as potential alternatives for the management of persistent insomnia because of their sedative properties. Mirtazapine was not initiated due to concerns regarding QT interval prolongation in the context of concomitant dasatinib treatment. Pharmacovigilance data have identified a positive signal for QT interval prolongation with mirtazapine [20]. Trazodone was not initiated because of concerns regarding QT interval prolongation in the context of concomitant oncological treatment. QTc prolongation, torsades de pointes, and other cardiac arrhythmias have been reported with trazodone, although at low incidence [21].

Buspirone was added as a non-benzodiazepine anxiolytic because of persistent anxiety symptoms. Buspirone is included among pharmacological treatment options for anxiety in patients with cancer, with a recommended starting dose of 7.5 mg twice daily and a maximum dose of 30 mg per day [22]. 

Due to the patient's complaints of anxiety attacks, bromazepam was prescribed on an as-needed basis. Benzodiazepines are effective in the acute treatment of anxiety disorders because of their rapid onset of action and are frequently used for short-term management. However, their long-term use is limited by the potential for abuse and dependence, and patients receiving benzodiazepines should be monitored for cognitive impairment, sedation, and falls, particularly when other sedating medications are used concomitantly [22]. In the present case, as antidepressant therapy and other psychotropic medications were adjusted and the patient participated in psychological and rehabilitation activities during hospitalisation, bromazepam was no longer required during the final weeks of treatment.

Phenibut was added to the treatment regimen because of persistent anxiety characterised by nervousness, inner restlessness, difficulty relaxing, and excessive worry. Phenibut is a GABAergic compound with anxiolytic and antinociceptive effects, acting primarily through GABA-B receptors and the α2δ subunit of voltage-gated calcium channels [23]. According to the Latvian Medicinal Product Register, phenibut is indicated for asthenic and anxiety-related neurotic states, including restlessness, anxiety, and fear; insomnia and nocturnal agitation in older adults; prevention of stress before surgical procedures; Ménière’s disease and dizziness associated with vestibular dysfunction of various etiologies; prevention of motion sickness (kinetosis); stuttering and tics in children; and as an adjunct in the management of alcohol withdrawal syndrome. For asthenic and anxiety-related neurotic states in adults, the recommended treatment course is two to three weeks, which may be extended to four to six weeks if clinically necessary [24]. Given the patient's concerns regarding medication dependence, phenibut was prescribed as a short-term, course-based treatment rather than for continuous use. According to the Latvian Medicinal Product Register, post-marketing experience with phenibut at therapeutic doses has not indicated a withdrawal syndrome; however, dependence may develop with doses exceeding the therapeutic range, and abrupt discontinuation after supratherapeutic use may result in a potentially severe withdrawal syndrome [24]. Therefore, in this patient, the potential risk of dependence was considered in the context of dosing and treatment duration, and particular caution was warranted regarding abrupt dose reduction or discontinuation. The dose was subsequently reduced from 250 mg twice daily to 125 mg twice daily at the patient's request because of concerns about medication dependence. Although the patient's anxiety symptoms improved during hospitalisation, the contribution of phenibut to this improvement cannot be established, as other pharmacological treatments were adjusted concurrently and psychological and multidisciplinary rehabilitation interventions were also provided.

Meldonium was initiated during hospitalisation as part of the patient's treatment regimen. According to the Latvian Medicinal Product Register, meldonium is indicated as part of combination therapy for reduced work capacity and physical overexertion, with a recommended dose of 500 mg daily in adults [25]. Evidence supporting the use of meldonium for cancer-related fatigue remains limited. However, a recently published clinical study in patients with metastatic renal cell carcinoma receiving systemic anticancer therapy found that meldonium was generally well tolerated and was associated with a reduction in treatment-related fatigue, particularly at a dose of 1,000 mg daily [26]. Given the patient’s concomitant oncological and psychotropic treatment, the safety profile and potential drug-drug interactions of meldonium were also considered. The Summary of Product Characteristics does not specifically describe interactions between meldonium and the patient's concomitant oncological or psychotropic medications. However, the absence of documented interactions does not exclude the possibility of clinically relevant interactions. The Summary of Product Characteristics notes that meldonium may potentiate the effects of certain antihypertensive agents, beta-blockers, nifedipine, glyceryl trinitrate, and peripheral vasodilators [25]. Rare adverse effects include agitation, sleep disturbances, palpitations, tachycardia, blood pressure changes, and ECG abnormalities [25]. No adverse effects attributable to meldonium were observed during hospitalisation. Meldonium was prescribed as a time-limited course of up to four weeks, with follow-up by the outpatient psychiatrist after discharge to reassess the need for continued treatment and monitor tolerability and potential adverse effects.

Patients with cancer experience insomnia, and management requires consideration of both non-pharmacological and pharmacological approaches. Management of insomnia in patients with cancer can be challenging, particularly in the context of polypharmacy. Hypnotics may be considered for short-term treatment of insomnia; however, evidence from randomised controlled trials in patients with cancer remains limited. Zopiclone, a non-benzodiazepine “Z-drug”, was used in the present case to address severe insomnia. Long-term use of hypnotics is generally discouraged because of adverse effects, tolerance, dependence and withdrawal insomnia [12].

Quetiapine was continued and subsequently titrated in the context of persistent insomnia. The dose adjustment was made taking into account the patient's good tolerability of quetiapine and her reluctance to switch to an alternative medication. Although quetiapine has been used off-label for insomnia in patients with cancer, evidence supporting its use in this population remains limited [27]. Although quetiapine also has QT-prolonging potential, its continuation and subsequent titration to 150 mg nightly were considered acceptable based on the normal baseline ECG, previous tolerability, and the anticipated clinical benefit for persistent insomnia and anxiety. In contrast, initiation of an additional psychotropic medication such as mirtazapine or trazodone was considered less favourable given the patient’s existing polypharmacy and the potential for cumulative QT-prolonging effects. An ECG performed prior to discharge showed no clinically significant abnormalities, with the QTc remaining within the normal range.

Dasatinib is primarily metabolised by the cytochrome P450 3A4 (CYP3A4) enzyme system, which is involved in the metabolism of numerous medications. Consequently, concomitant use of medications that inhibit or induce CYP3A4 may alter dasatinib exposure. Strong CYP3A4 inhibitors may increase dasatinib exposure, whereas CYP3A4 inducers may reduce its plasma concentrations and potentially compromise treatment efficacy [18]. 

Psychiatric adverse reactions have also been reported during dasatinib treatment, with depression and insomnia classified as common adverse reactions and anxiety as an uncommon adverse reaction [18]. In the present case, the patient's psychiatric deterioration occurred in the context of multiple potentially contributing factors, including her pre-existing recurrent depressive disorder, the underlying haematological disease and its treatment, and the need for concomitant oncological and psychotropic medications. According to the patient's reported history, the deterioration in depressive and anxiety symptoms preceded the initiation of dasatinib. Therefore, a direct causal relationship between dasatinib and the patient's psychiatric symptoms could not be established. These considerations were taken into account when selecting and adjusting psychotropic treatment, with treatment decisions individualised according to the overall clinical presentation, potential drug-drug interactions, and the need to balance efficacy and safety during concomitant dasatinib therapy.

Psychotropic medications were therefore selected and adjusted based on the patient's clinical symptoms, treatment response, potential drug-drug interactions, and individual treatment preferences. The patient's expressed concerns regarding medication changes, including concerns about potential dependence and her reluctance to switch from well-tolerated medications, were also taken into consideration when making subsequent treatment decisions.

The relatively frequent medication adjustments during hospitalisation may have limited the ability to distinguish the effects of individual medications on the observed clinical improvement, particularly given the concurrent pharmacological and non-pharmacological interventions. However, the inpatient setting allowed for close monitoring of the patient's clinical status, treatment tolerability, and response to medication, facilitating timely adjustments when clinically indicated.

In addition to pharmacological treatment, the patient received psychological interventions with a CBT and supportive therapy orientation as part of the multidisciplinary psychiatric rehabilitation programme. In patients with cancer and clinically significant depression, multidisciplinary collaborative care integrating pharmacological and psychological interventions is more effective than usual care in reducing depressive symptoms [28]. In the present case, the multidisciplinary approach, including psychological interventions and psychiatric rehabilitation therapies, complemented pharmacological treatment, alongside gradual psychological and functional improvement.

During hospitalisation, the patient demonstrated gradual clinical improvement, with a reduction in anxiety and depressive symptoms and improvement in sleep. The patient also reported an overall improvement in her emotional state and subjective well-being. The patient's marked anxiety and repeated concerns regarding treatment changes influenced the therapeutic process. Establishing a sense of safety and addressing her concerns required considerable time and patience. Her willingness to communicate medication concerns, particularly her concerns about potential dependence, and her active participation in treatment decisions influenced subsequent adjustments of the pharmacological regimen. Patient engagement and adherence were, therefore, important factors in individualising treatment.

## Conclusions

This case highlights the importance of individualised psychopharmacological treatment and multidisciplinary collaboration in the management of patients with psychiatric and oncological comorbidities. The patient's clinical improvement occurred during hospitalisation, when pharmacological treatment, psychological support, and psychiatric rehabilitation were provided concurrently. Careful consideration of potential drug-drug interactions, treatment tolerability, and ongoing safety monitoring is particularly important in patients receiving concomitant oncological and psychotropic medications. Further development of integrated psychiatric and oncological care in Latvia could contribute to more systematic recognition and management of psychiatric symptoms in patients undergoing cancer treatment.
